# Prognostic significance of high-sensitivity cardiac troponin in patients with heart failure with preserved ejection fraction

**DOI:** 10.1007/s00380-019-01393-2

**Published:** 2019-03-30

**Authors:** Sho Suzuki, Hirohiko Motoki, Masatoshi Minamisawa, Yukari Okuma, Wataru Shoin, Takahiro Okano, Kazuhiro Kimura, Soichiro Ebisawa, Ayako Okada, Koichiro Kuwahara

**Affiliations:** Department of Cardiovascular Medicine, Shinshu University School of Medicine, Asahi 3-1-1, Matsumoto, 3908621 Nagano Japan

**Keywords:** Heart failure with preserved ejection fraction, High-sensitivity troponin T, Prognosis

## Abstract

The aim of this study was to investigate the prognostic significance of high-sensitivity troponin T (hs-TnT) in patients with heart failure (HF) with preserved ejection fraction (HFpEF). We enrolled consecutive patients admitted to Shinshu University Hospital for HF treatment between July 2014 and March 2017 and stratified them into HF with reduced ejection fraction and HFpEF groups (left ventricular ejection fraction, < 50% and  ≥ 50%, respectively). Hs-TnT was evaluated at discharge, and patients were prospectively monitored for all-cause mortality, non-fatal myocardial infarction, stroke, and HF hospitalization. In 155 enrolled patients (median age 76 years), during a median follow-up of 449 days, 60 experienced an adverse event. Hs-TnT was significantly higher in patients with adverse events than in those without in HFpEF (*p* = 0.003). Hs-TnT did not significantly correlate with age, sex, hemoglobin, albumin, eGFR, or BNP. In Kaplan–Meier analysis, high hs-TnT predicted a poor prognosis in HFpEF (*p* = 0.003). In multivariate Cox regression analysis, hs-TnT levels independently predicted adverse events in HFpEF (*p* = 0.003) after adjusting for age and eGFR [HR, 1.015 (95% CI, 1.005–1.025), *p* = 0.004], and age and BNP [HR 1.016 (95% CI 1.005–1.027), *p* = 0.005]. Elevated hs-TnT at discharge predicted adverse events in HFpEF.

## Introduction

Approximately half of all patients hospitalized with heart failure (HF) have a normal left ventricular ejection fraction (LVEF), a condition known as HF with preserved ejection fraction (HFpEF) [1]. The mortality rate of patients with HFpEF is reportedly comparable to that of those with HF with reduced ejection fraction (HFrEF) [2]. Thus, tools for risk-stratification in hospitalized patients with HFpEF are needed to improve the management of these patients.

B-type natriuretic peptide (BNP) and N-terminal pro-BNP have been used for HF diagnosis, prognosis, and management, as established biomarkers. Additionally, there has been increased interest in the utility of troponin, a biomarker of myocardial necrosis, to predict prognosis in patients with HF [3]. Current HF guidelines recommend troponin measurement on hospital admission to establish prognosis in cases of acutely decompensated HF [4, 5]. However, these recommendations are based on studies that included not only patients with HFpEF, but also those with HFrEF. Several studies have demonstrated a consistent association between elevated troponin and adverse clinical outcomes in patients with HFrEF [6, 7]. On the other hand, the prognostic value of troponin in HFpEF patients is not well-established.

The introduction of high-sensitivity assays has allowed the accurate detection of very low levels of circulating cardiac troponins in stable HF [8]. Compared to the cardiac troponin T (cTnT), the high-sensitivity troponin T (hs-TnT) assay is expected to demonstrate superior clinical performance in the setting of cardiovascular events. While a few previous investigators evaluated the prognostic value of troponin in HFpEF retrospectively, these studies assessed the efficacy of cardiac troponin I (cTnI) or a combination of cTnI and cTnT [9, 10]. Therefore, we aimed to investigate the prognostic significance of hs-TnT in patients hospitalized for decompensated HFpEF.

## Materials and methods

### Study population

This study enrolled consecutive patients admitted to Shinshu University Hospital for HF treatment between July 2014 and March 2017 with the exception of acute coronary syndrome. Informed consent was obtained after the HF treatment. Then, patients were enrolled at the compensated state of HF before discharge. We recorded baseline clinical characteristics including age, sex, medical history, HF etiology, major risk factors for coronary heart disease (hypertension, smoking, diabetes, and dyslipidemia), comorbidities, and HF medications in all registered patients. We performed blood tests and echocardiography at discharge.

The diagnosis of HF was made by symptoms, physical examinations, chest X-rays, echocardiography, and blood tests. The diagnosis of acute coronary syndrome was made by treating clinicians using symptoms, electrocardiograms, echocardiography, blood tests, chest X-rays, and available coronary angiograms. Patients underwent a registration examination when they became clinically stable following HF treatment.

We performed transthoracic echocardiography using standardized equipment (Vivid E9 Ultrasound Machine; GE Healthcare, Chicago, IL, US) in compliance with the recommendations of the American Society of Echocardiography [11]. The biplane modified Simpson’s method was used to measure LVEF. We stratified patients into HFrEF (LVEF < 50%) and HFpEF (LVEF ≥ 50%) subgroups according to their LVEF at discharge. We measured hs-TnT at discharge using an electrochemiluminescence immunoassay and a Cobas e411 analyzer (Roche Diagnostics GmBH, Mannheim, Germany). The measurement range of the hs-TnT assay was 3–10,000 ng/L with a coefficient of variation of 15% at the level of 3 ng/L.

The Shinshu University School Hospital Ethics Committee approved the study protocol. The investigation confirms the principles outlined in the Declaration of Helsinki.

### Follow-up

Patients were prospectively monitored for major adverse cardiac events (all-cause mortality, non-fatal myocardial infarction, non-fatal stroke, and HF hospitalizations) through scheduled telephone follow-up, and incidents were validated by chart review.

### Statistical analysis

Continuous variables are summarized as means ± standard deviation if normally distributed and as medians with interquartile range if non-normally distributed. Normality was assessed by the Shapiro–Wilk *W* test. Comparisons of baseline characteristics were made with a contingency table for categorical variables, *t* test for normally distributed continuous variables, and either the Wilcoxon or Mann–Whitney test for non-normally distributed continuous variables. Spearman’s rank correlation method was used as a nonparametric measure of association between hs-TnT and clinical and laboratory indices. The optimal receiver operating characteristic (ROC) curve cutoff value for prediction of adverse clinical events was chosen as the value maximizing sensitivity and specificity. Kaplan–Meier survival plots were calculated from baseline to time of adverse event and compared using the log-rank test. Cox proportional hazards analysis was used to evaluate the independent prognostic utility of hs-TnT. The covariates used were age, sex, estimated glomerular filtration rate (eGFR), hemoglobin, albumin, and BNP. A *p* value < 0.05 was considered statistically significant. The statistical analyses were performed using SPSS Statistics for Windows, Version 24 (IBM Corp., Armonk, NY, US).

## Results

### Study population

We enrolled 155 patients (mean age, 76; male, 62%). Sixty-four (41%) patients had HFpEF, and 91 (59%) had HFrEF. Table 1 shows baseline patient characteristics stratified by HF group. In terms of comorbidities, 88 (57%) patients had atrial fibrillation, which was relatively high. Among them, 4 patients (3 patients in HFpEF, 1 patient in HFrEF) had a history of previous catheter ablation. Other patients were treated by anticoagulant therapy, and either medical rate control or rhythm control. In total, 11 patients (2 patients in HFpEF, 9 patients in HFrEF) underwent percutaneous coronary intervention during hospitalization due to newly diagnosed coronary artery disease. Compared to those who did not develop adverse events, patients who did were older and had higher hs-TnT in the HFpEF (36 [20–66] ng/L vs. 21 [15–32] ng/L, p = 0.003) and HFrEF (40 [29–71] ng/L vs. 27 [16–50] ng/L, p = 0.005) groups. There were no significant differences in BNP levels in patients with and without an adverse event. In the HFpEF group, albumin, hemoglobin, and eGFR were lower in patients who developed adverse events than in those who were event-free. However, there were no significant correlations between hs-TnT and these clinical indices (Table 2).

**Table 1:** Baseline characteristics in patients with heart failure

Variable	Overall population (*n* = 155)	HFpEF (*n* = 64)			HFrEF (*n* = 91)		
		Adverse cardiac events			Adverse cardiac events		
		Yes (*n* = 28)	No (*n* = 36)	*p* value	Yes (*n* = 32)	No (*n* = 59)	*p* value
Age (years) [range]	76 [67–84]	84 ± 11	75 ± 11	0.002	80 [70–84]	69 [58–77]	< 0.001
Male sex, *n* (%)	96 (62)	16 (57)	16 (44)	0.313	22 (69)	42 (71)	0.808
BMI	21.0 [19.0–24.2]	20.5 [18.7–24.2]	22.1 [19.3–25.6]	0.223	20.2 [17.9–22.7]	21.0 [19.2–24.1]	0.16
Systolic blood pressure, mmHg	113 ± 17	119 ± 17	117 ± 18	0.568	107 ± 15	110 ± 17	0.423
NYHA class III or IV, *n* (%)	40 (26)	9 (32)	5 (14)	0.08	11 (34)	15 (25)	0.367
Ischemic etiology, *n* (%)	50 (32)	7 (25)	8 (22)	0.795	16 (50)	19 (32)	0.096
Hypertension, *n* (%)	82 (53)	17 (61)	22 (61)	0.974	11 (34)	32 (54)	0.07
Dyslipidemia, *n* (%)	50 (32)	6 (21)	11 (31)	0.412	13 (41)	20 (34)	0.524
Diabetes mellitus, *n* (%)	53 (34)	6 (21)	10 (28)	0.561	17 (32)	20 (34)	0.075
Atrial fibrillation, *n* (%)	88 (57)	18 (64)	24 (67)	0.842	19 (59)	27 (46)	0.215
Medication							
Antiplatelet, *n* (%)	73 (47)	12 (43)	14 (39)	0.748	21 (66)	26 (44)	0.049
Anticoagulant, *n* (%)	98 (63)	17 (61)	25 (69)	0.466	20 (63)	36 (61)	0.89
ACE-I, *n* (%)	89 (57)	10 (36)	17 (47)	0.355	24 (75)	38 (64)	0.3
ARB, *n* (%)	38 (25)	8 (28)	12 (22)	0.683	5 (16)	13 (22)	0.464
ACE-I and/or ARB, *n* (%)	126 (81)	18 (64)	28 (78)	0.234	29 (91)	51 (86)	0.559
Beta-blocker, *n* (%)	111 (72)	12 (43)	22 (61)	0.147	26 (81)	51 (86)	0.512
MRA, *n* (%)	92 (59)	17 (61)	21 (58)	0.847	17 (53)	37 (63)	0.374
Loop diuretic, *n* (%)	127 (82)	24 (86)	29 (81)	0.587	29 (91)	45 (76)	0.093
Tolvaptan, *n* (%)	37 (24)	6 (21)	6 (17)	0.628	10 (31)	15 (25)	0.552
Laboratory data							
Alb (g/dL)	3.5 [3.3–3.9]	3.4 [3.2–3.5]	3.6 [3.3–3.8]	0.006	3.6 ± 0.5	3.6 ± 0.5	0.97
Hb (g/dL)	11.7 [10.4–13.6]	10.7 ± 1.4	11.9 ± 1.8	0.004	11.7 [10.3–12.9]	12.9 [10.8–14.4]	0.063
HbA1c (%)	6.0 [5.7–6.4]	6.0 [5.5–6.0]	5.8 [5.7–6.2]	0.169	6.2 [5.9–6.8]	6.1 [5.8–6.6]	0.306
eGFR (mL/min/1.73 m^2^)	45 [31–58]	35 [28–48]	51 [36–69]	0.005	38 [25–57]	46 [39–61]	0.085
BNP, pg/mL	269 [140–479]	240 [160–376]	134 [61–302]	0.093	514 [241–649]	291 [177–499]	0.055
hs-TnT, ng/L	30 [19–50]	36 [20–66]	21 [15–32]	0.003	40 [29–71]	27 [16–50]	0.005
Echocardiographic data							
LVEF (%)	46 ± 16	59 [53–70]	60 [55–64]	0.901	35 [28–43]	35 [29–43]	0.816
LAD (mm)	49 [43–55]	51 [45–62]	50 [45–54]	0.253	50 [45–56]	47 [42–52]	0.043
LVEDV Index (mL/m^2^)	68[47–87]	48 [38–57]	42 [38–56]	0.967	80 [69–106]	80 [66–95]	0.461
LVESV Index (mL/m^2^)	36 [21–58]	19 [15–24]	18 [15–24]	0.877	55 [38–77]	48 [36–66]	0.348
LVDd (mm)	54 ± 9	47 ± 6	48 ± 6	0.365	59 ± 8	57 ± 9	0.444
LVDs (mm)	39 [31–48]	30 ± 5	31 ± 6	0.411	47 ± 10	47 ± 10	0.793
Severe AS	4 (3)	0 (0)	1 (4)	0.437	2 (6)	1 (2)	0.282
Severe AR	3 (2)	1 (4)	1 (3)	0.688	0 (0)	1 (2)	0.648
Severe MR	13 (8)	1 (4)	6 (17)	0.096	4 (13)	2 (3)	0.111
Mitral E/A ratio	1.0 [0.7–1.8]	1.33 ± 0.36	1.60 ± 0.86	0.264	0.92 [0.67–1.65]	0.92 [0.65–1.83]	0.885
Mitral DT (msec)	163 [128–212]	193 [142–225]	166 [147–218]	0.843	148 [115–207]	153 [128–244]	0.45
Mean E/e' ratio	13.2 [10.3–19.6]	9.7 [9.4–14.1]	13.1 [10.1–14.3]	0.383	16.5 [13.3–31.0]	14.2 [12.5–25.5]	0.662

Values are mean ± SD, median [interquartile range], or *n* (%)

*ACE-I *Angiotensin-converting enzyme inhibitor, *Alb *albumin, *AR *aortic regurgitation, *ARB *angiotensin-receptor blocker, *AS *aortic stenosis, *BMI *body mass index, *BNP *B-type natriuretic peptide, *Dd *diastolic dimension, *Ds *systolic dimension, *DT *deceleration time, *E *peak early mitral inflow velocity, *e’ *peak early diastolic velocity at the mitral annulus, *EDV *end-diastolic volume, *EF *ejection fraction, *eGFR *estimated glomerular filtration rate, *ESV *endo-systolic volume, *Hb *hemoglobin, *HbA1c *hemoglobin A1c, *HFpEF *heart failure with preserved ejection fraction, *HFrEF *heart failure with reduced ejection fraction, *hs-TnT *high-sensitivity troponin T, *LAD *left atrial dimension, *LV *left ventricular, *MR *mitral regurgitation, *MRA *mineralocorticoid receptor antagonist, *NYHA *New York Heart Association

**Table 2:** Univariate Spearman’s rank correlations between high-sensitivity troponin T and clinical indices in patients with heart failure with preserved ejection fraction

Variable	Spearman’s *r*	*p* value
Age (years)	0.093	0.446
Sex	− 0.202	0.109
Hb (g/dL)	− 0.188	0.137
Alb (g/dL)	− 0.053	0.678
eGFR (mL/min/1.73 m^2^)	− 0.141	0.267
BNP (pg/mL)	0.182	0.175
LAD (mm)	0.176	0.164
LVEDV Index (mL/m^2^)	− 0.006	0.965
LVESV Index (mL/m^2^)	0.041	0.753
Mean E/e′ ratio	0.240	0.568

*Alb* Albumin, *BNP *B-type natriuretic peptide, *E *peak early mitral inflow velocity, *e′ *peak early diastolic velocity at the mitral annulus, *EDV *end-diastolic volume, *EF *ejection fraction, *eGFR *estimated glomerular filtration rate, *ESV *endo-systolic volume, *Hb *hemoglobin, *LAD *left atrial dimension, *LV *left ventricular

### The prognostic impact of hs-TnT

During a median follow-up of 449 days [interquartile range: 260–780], 60/155 (39%) patients experienced an adverse event (all-cause mortality, 31; non-fatal myocardial infarction, 2; non-fatal stroke, 2; HF hospitalization, 45). Adverse events occurred in 28 (44%) HFpEF group patients (all-cause mortality, 12; non-fatal myocardial infarction, 1; non-fatal stroke, 1; HF hospitalization, 23) and 32 (35%) HFrEF group patients (all-cause mortality, 19; non-fatal myocardial infarction, 1; non-fatal stroke, 1; HF hospitalization, 22). There were 3 (4%) patients in HFpEF, and 7 (8%) patients in HFrEF who had elevated hs-TnT over the upper reference limit of the troponin assay (i.e., hs-TnT ≥ 100 ng/L).

**Fig. 1:**
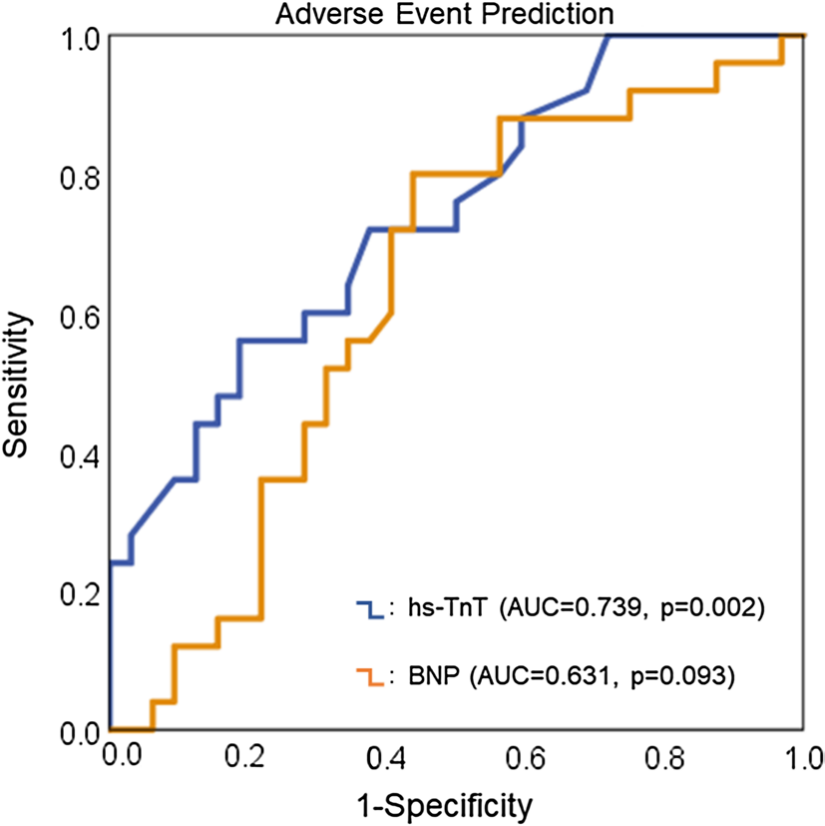
Receiver operating characteristic curve for the prediction of adverse cardiac events in patients with heart failure with preserved ejection fraction—the greatest area under the high-sensitivity troponin T (hs-TnT) receiver operating characteristic (ROC) curve (AUC)—occurs at the optimal cutoff point of 25.5 ng/L. Blue line, hs-TnT; orange line, B-type natriuretic peptide (BNP)

**Fig. 2:**
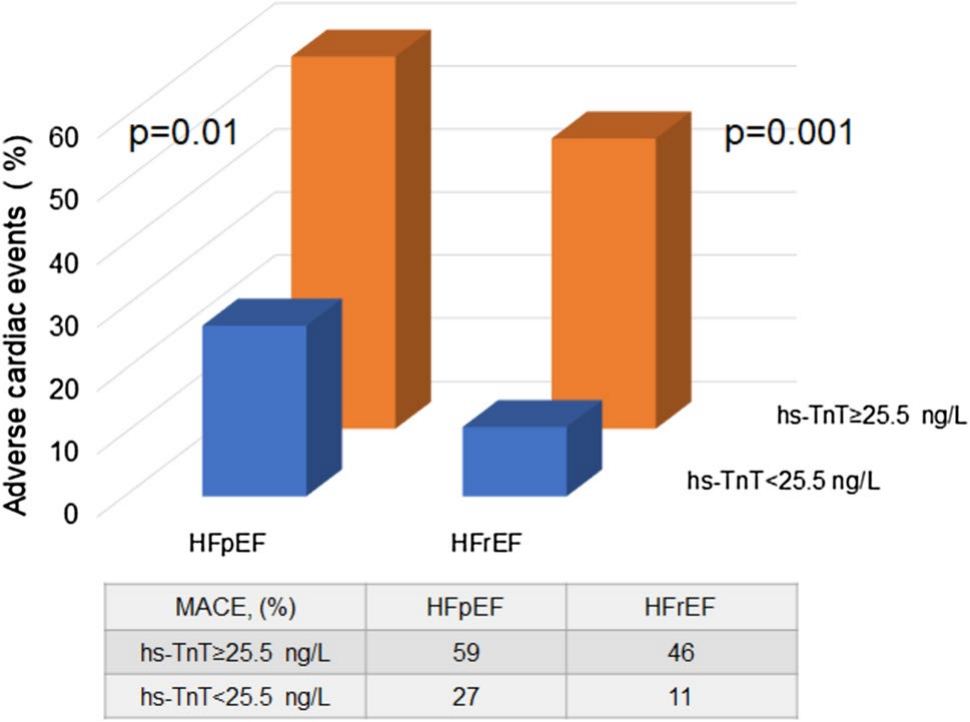
Incidence of cardiac events according to high-sensitivity troponin T level—elevated high-sensitivity troponin T (hs-TnT  ≥ 25.5 ng/L)—was related to an increased risk of major adverse cardiac events (all-cause mortality, non-fatal myocardial infarction, non-fatal stroke, and HF hospitalizations) in groups with heart failure with preserved ejection fraction (HFpEF) and heart failure with reduced ejection fraction (HFrEF)

**Fig. 3:**
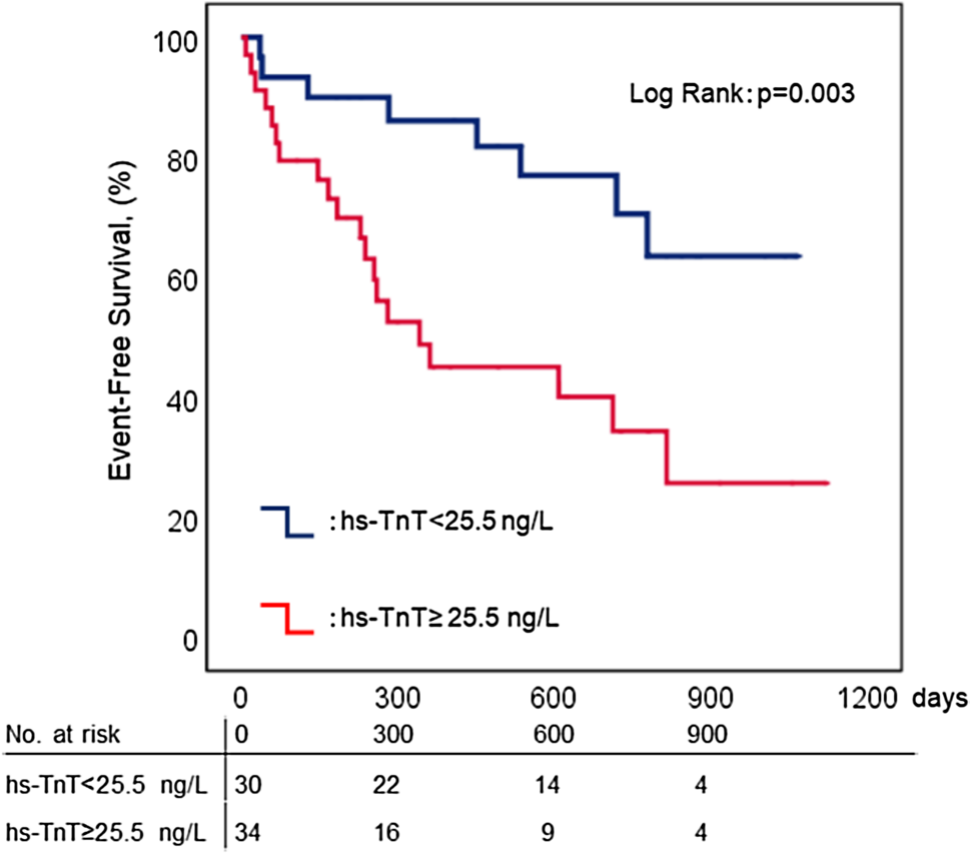
Kaplan–Meier analysis of high-sensitivity troponin T in patients with heart failure with preserved ejection fraction—elevated high-sensitivity troponin T (hs-TnT  ≥ 25.5 ng/L)—predicted adverse cardiac events (red line). Blue line, hs-TnT  < 25 ng/L

In our hs-TnT ROC analysis, the area under the curve was greatest at an optimal cutoff point of 25.5 ng/L in the HFpEF cohort (Fig. 1). The area under the curve was greater in hs-TnT than in BNP assays. High hs-TnT levels were related to an increased risk of adverse events in both HFpEF and HFrEF groups (Fig. 2). In Kaplan–Meier analysis, hs-TnT ≥ 25.5 ng/L predicted adverse events in the HFpEF group (Fig. 3). In multivariate Cox proportional hazards analysis, hs-TnT ≥ 25.5 ng/L predicted adverse events after adjustment for age, sex, eGFR, hemoglobin, albumin, and BNP in patients with HFpEF (Table 3).

**Table 3:** Multivariable Cox proportional hazards analysis in heart failure with preserved ejection fraction

Variables	HR (95% CI)	*p* value
hs-TnT adjusted for		
Age, sex	1.015 (1.005–1.025)	0.004
Age, eGFR	1.014 (1.005–1.024)	0.004
Age, Hb	1.015 (1.005–1.025)	0.003
Age, Alb	1.017 (1.006–1.028)	0.002
Age, BNP	1.016 (1.005–1.027)	0.005

*Alb *Albumin, *BNP *B-type natriuretic peptide, *CI *confidence interval, *eGFR *estimated glomerular filtration rate, *Hb *hemoglobin, *HR *hazard ratio, *hs-TnT *high-sensitivity troponin T

## Discussion

In this study, we identified a significant association between elevated hs-TnT at discharge and adverse events in patients hospitalized with decompensated HFpEF. This association was independent of other well-established laboratory risk predictors, including BNP. A recent study reported the value of hs-TnT and high-sensitivity troponin I for predicting adverse events in stable HFpEF and HFrEF [12]. However, to our knowledge, no prior studies investigated the use of hs-TnT in Japanese elderly patients with decompensated HFpEF. In this study, we demonstrated that hs-TnT has prognostic significance in this population. This finding has important clinical implications and suggests that hs-TnT is a useful risk-stratification tool in cases of HFpEF. While several reports showed the prognostic significance of troponin T at admission [9, 10, 12], our study indicates that the addition of hs-TnT measurement at discharge could identify patients with HFpEF who require aggressive therapy and close outpatient follow-up. When talking about BNP, some studies report that the BNP value at discharge is a more accurate predictor of mortality in HF patients than that of admission or admission-to-discharge reduction [13]. Similarly, hs-TnT at discharge may be a better tool for prognostic prediction than troponin T at admission.

Myocyte cell death is the main pathology of troponin elevation in acute coronary syndrome. In HFrEF, the pathophysiological mechanism of myocardial injury and troponin elevation include subendocardial ischemia, neurohormonal activation, inflammatory cytokine release, altered calcium handling, oxidative stress, and increased wall stress [14]. Under these mechanisms, cardiac troponin shows significant relation with adverse cardiac events.

On the other hand, the fundamental pathophysiological mechanism of troponin elevation in HFpEF remains unclear. In HFpEF, ventricular diastolic dysfunction (impaired relaxation and increased diastolic stiffness) is typically present at rest or induced by stress (e.g., exercise, tachycardia, or hypertension) [15, 16]. Endothelial dysfunction, arterial stiffening, and increased ventricular systolic stiffness are also common [17]. Recently, systemic microvascular endothelial inflammation related to comorbid conditions has been proposed as another mechanism leading to myocardial inflammation and fibrosis and cardiomyocyte signaling pathway alterations. These alterations promote cell remodeling and dysfunction [18, 19], microvascular dysfunction and rarefaction in cardiac and skeletal muscle [20–23], and increases in oxidative stress [17]. From these backgrounds, troponin elevation in HFpEF might indicate the microvascular endothelial inflammation leading to myocardial death and subsequent fibrosis. However, this hypothesis is only speculative, and further studies are needed.

Our study had several limitations. First, we included a small number of patients taken from a single center. The number of patients is not enough to assess the prognostic value of hs-TnT, and the consecutive patients in this study represents a very selected cohort. Further research in a large cohort is necessary to verify our findings. Second, although elevated hs-TnT was independently associated with adverse events after adjusting for age and eGFR, HFpEF patients who had poor prognosis were older, and had impaired renal function. It is clear that these two indices are significantly associated with worse outcomes. To use hs-TnT as a risk-stratification tool in Japanese elderly patients, association with cardiac death or HF re-admission should be investigated in larger studies. Third, only a single measure of hs-TnT was available in each case, and serial hs-TnT levels were not evaluated. However, we measured hs-TnT at discharge when patients were in a stable phase of heart failure, and serial changes were expected to be rather small. Forth, patients who died in the hospital were not included in our study, and the most severe HF cases may have been excluded as a result. Finally, patients who underwent percutaneous coronary intervention during hospitalization were included in this study, which might have influenced in the hs-TnT at discharge. However, the median hs-TnT of these patients was 31 [24–45] ng/L, which had no difference compared to the total population (30 [19–50] ng/L, *p* = 0.557).

In conclusion, elevated hs-TnT was independently associated with adverse cardiac events in hospitalized patients with decompensated HFpEF. Our findings suggest that hs-TnT may be a useful risk-stratification tool in this population. Further studies are needed to identify the multiple mechanisms leading to troponin T release in decompensated HFpEF.
